# Carbon-Based Nanocomposite Smart Sensors for the Rapid Detection of Mycotoxins

**DOI:** 10.3390/nano11112851

**Published:** 2021-10-26

**Authors:** Xiaoli Ma, Xinbo Li, Wenrui Zhang, Fanxing Meng, Xin Wang, Yanan Qin, Minwei Zhang

**Affiliations:** 1 Xinjiang Key Laboratory of Biological Resources and Genetic Engineering, College of Life Science and Technology, Xinjiang University, Urumqi 830046, China; xlm87@xju.edu.cn (X.M.); nylxb97@163.com (X.L.); 18419967329@163.com (W.Z.); k_h@stu.xju.edu.cn (F.M.); 2 Synthetic Biology Laboratory, School of Future Technology, Xinjiang University, Urumqi 830046, China; 3 Institute of Translational Medicine, The First Hospital of Jilin University, Changchun 130061, China; xinwang-nn@hit.edu.cn

**Keywords:** graphene, carbon nanotubes, nanocomposites, smart sensors, mycotoxins

## Abstract

Carbon-based nanomaterials have become the subject of intensive interest because their intriguing physical and chemical properties are different from those of their bulk counterparts, leading to novel applications in smart sensors. Mycotoxins are secondary metabolites with different structures and toxic effects produced by fungi. Mycotoxins have low molecular weights and highly diverse molecular structures, which can induce a spectrum of biological effects in humans and animals even at low concentrations. A tremendous amount of biosensor platforms based on various carbon nanocomposites have been developed for the determination of mycotoxins. Therefore, the contents of this review are based on a balanced combination of our own studies and selected research studies performed by academic groups worldwide. We first address the vital preparation methods of biorecognition unit (antibodies, aptamers, molecularly imprinted polymers)-functionalized carbon-based nanomaterials for sensing mycotoxins. Then, we summarize various types of smart sensors for the detection of mycotoxins. We expect future research on smart sensors to show a significant impact on the detection of mycotoxins in food products.

## 1. Introduction

As a secondary metabolite of a variety of fungal species found worldwide, mycotoxins exposed to foodstuffs not only cause significant health risks to humans (e.g., cancers, teratogenicity, hepatotoxicity, and immunotoxicity) but also cause severe economic losses [1,2,3,4]. To date, hundreds of mycotoxins have been found and sorted into different categories, with aflatoxins (AFs), fumonisins (FMs), zearalenone (ZEN), ochratoxins (OTs), and trichothecenes (TCTs) as the main and the most commonly occurring and toxicologically recognized classes [1,3,5]. Among them, aflatoxin B_1_ (AFB_1_) has been classified as a potent carcinogen to humans, while fumonisin and ochratoxin A (OTA) is possibly carcinogenic in humans. Due to the hazard of mycotoxins, they have aroused widespread concern with regards to global security [6]. Therefore, reliable and accurate detection means are necessary.

At present, conventional detection methods for mycotoxins are chromatographic methods, which include thin-layer chromatography (TLC), high-performance liquid chromatography (HPLC), gas chromatography (GC), and liquid chromatography-mass spectrometry (LC–MS). There are also immunological methods, including enzyme-linked immunosorbent assay (ELISA) and immunochromatography (ICA) [7,8,9,10]. Although these analytical methods are sensitive and selective towards mycotoxin detection, they normally require expensive instruments, sophisticated operation, complex preprocessing, and large time consumption. Therefore, the rapid analysis of mycotoxin development is becoming increasingly important. At present, the detection of mycotoxins by electrochemical technology is increasingly widespread [11,12,13].

Sensors, portable analytical facilities utilizing biorecognition units for the accurate identification of target analytes on the transducer interface, have been developed as ideal alternatives for efficient, rapid, and in situ mycotoxin detection [14]. In recent years, the selectivity and sensitivity of sensors have been obviously improved due to the integration of nanotechnology in the construction of sensors [15,16]. Various nanomaterials and their composites, such as gold nanoparticles (Au NPs), silver nanoparticles (Ag NPs), carbon nanotubes (CNTs), graphene, and other carbon nanomaterial metal/metal oxide nanoparticle composites, have been exploited for their excellent electrical/optical/catalytic properties in the design strategy of sensors, which offers great improvement in the sensitivity of sensors by increasing signal production. In particular, carbon nanomaterials have their own unique advantages, such as a high specific surface area, excellent electrical transmission ability, good biocompatibility, and easy functionalization, has and they have become promising materials for the detection of mycotoxins [17,18,19,20,21].

**Figure 1:**
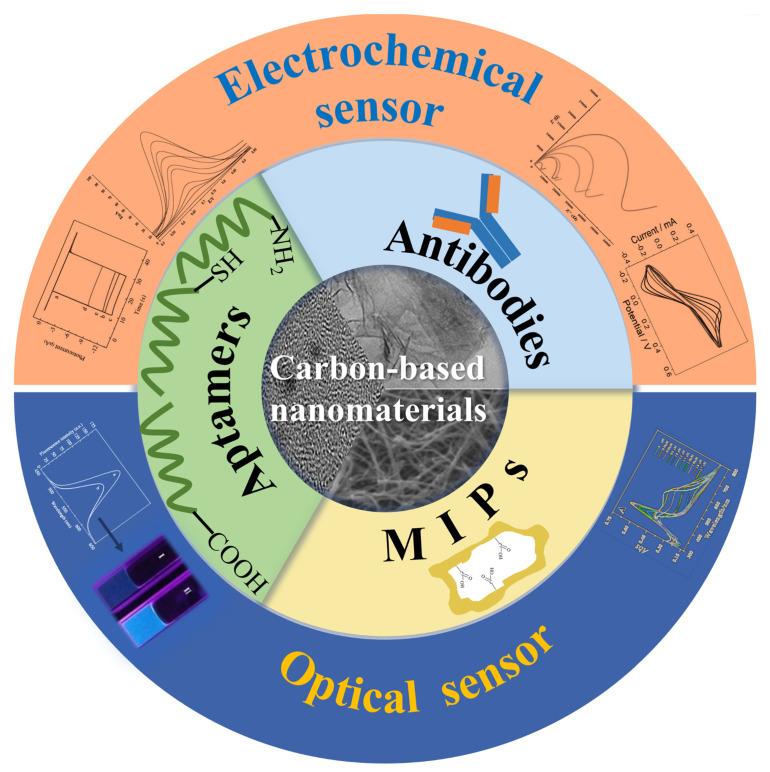
Functionalization of Carbon nanomaterials and its application of mycotoxins detection.

Herein, the present review first introduces various carbon nanomaterials (CNMs) and their functionalization by surface structures and different biorecognition units, such as antibodies, aptamers, and molecularly imprinted polymers (MIPs), for the detection of mycotoxins. Then, we summarize the recent developments of CNM sensors for mycotoxin detection. Finally, we discuss current challenges and provide a vision of the potential opportunities for mycotoxin detection in the hope of providing useful inspiration for researchers in the fields of food safety. Figure 1 outlines the interest and focus of the present review.

## 2. Carbon-Based Functional Nanomaterials

The unique characteristics of carbon and its allotropes are attributed to their sp, sp^2^, and sp^3^ hybridization [22]. The ratio of sp/sp^2^/sp^3^ hybridization in carbon nanomaterials determines the formation of flat 2D nanomaterials (graphene and its derivatives), hollow 1D nanomaterials (carbon fibers and CNTs), and closed 0D nanomaterials (graphene quantum dots (GQDs), carbon quantum dots (CQDs) and carbon spheres). In addition, this ratio also determines other properties of carbon nanomaterials, including magnetic properties, electrical properties, chemistry, and structural strength, which contribute to the unique advantages of different carbon nanomaterials in different applications [23,24]. The considerable specific superficial area of carbon materials could increase the quantities of bioactive molecules immobilized, increase the reaction sites of bioactive substances, improve electrical conductivity, and enhance responsiveness. To give full play to the advantages of these carbon materials in detection, it is necessary to modify the surface of carbon materials.

Surface modification of carbon nanomaterials is one of the key steps in the development of high-efficiency electrochemical sensors to achieve excellent performance. The performance of the smart sensor primarily depends on the identification elements. Various types of biorecognition units are modified on the electrode surface of the smart biosensor. Biorecognition units, such as antibodies, aptamers, and MIPs, have high specificity and selectivity for target analyte recognition. Combining a biorecognition unit with smart sensors could yield unimaginably superior results.

### 2.1. Antibody-Functionalized CNMs

Due to the selectivity, antibodies are combined with nanomaterials for the detection of mycotoxins. Carbon nanomaterials are widely used in mycotoxin sensors because of their easy surface modification and large specific surface area, which promote binding with antibodies. Recently, several efforts have been devoted to immobilizing specific antibodies on the electrode surface through covalent binding, self-assembly techniques, and electrostatic adsorption for the highly efficient detection of various mycotoxins.

**Figure 2:**
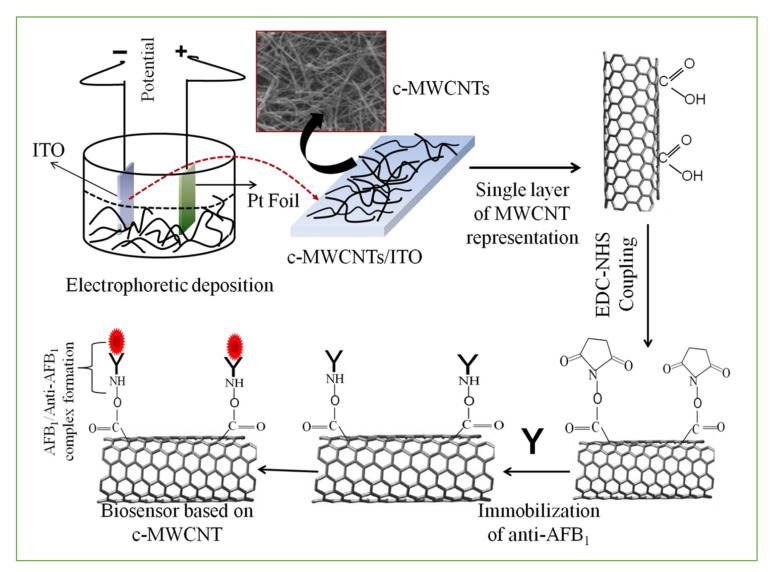
Schematic representation of c-MWCNTs based biosensor for aflatoxin B1 detection. Reprinted with permission from [25]. Copyright 2013 Elsevier.

Covalent binding is a simple, easy-to-operate method for the steady immobilization of antibodies on the surface of electrodes for building a sensor based on a specific group reaction, such as the carboxyl groups of nanomaterials such as CNTs and GO with the amine groups of the proteins, including antibodies. For example, the NH_2_ groups of antibodies were covalently bound to the COOH terminus of CNTs via strong amide bond (CO-NH) formation. A large number of carboxylic acid groups were formed on the surface of these carbon nanomaterials treated with high concentrations of HNO_3_/H_2_SO_4_. The carboxylated carbon nanomaterial composite electrode was mainly activated using N-ethyl-N’-(3-dimethylaminopropyl carbodiimide) (EDC) as the coupling agent and N-hydroxysuccinimide (NHS) as the activator. Then, antibodies were tightly attached to the electrode surface for mycotoxin detection (Figure 2) [25,26].

Moreover, antibody-modified carbon-based electrodes use a self-assembly technique and electrostatic adsorption. To construct the AFB_1_ sensor, anti-AFB_1_ was immobilized onto an Au nanodot/rGO nanosheet/ITO electrode based on a self-assembly technique in which the Au nanodots acted as anchoring points for anti-AFB_1_ [27]. Similarly, the antibody was linked with gold on the electrode as a binding site. The Au NP/WS_2_/MWCNT/GCE electrode was coated with anti-AFB1 nanobodies via the interaction between amine/sulfhydryl groups of nanobodies and Au NPs. In this study, an electrochemical immunosensor based on the signal amplification strategy of the AFB_1_-hybridization chain reaction to rapidly and sensitively determine AFB_1_ was constructed, whose applicability was verified by corn samples [28]. Electrostatic adsorption is a one-step method without introducing additional impurities, which is based on the zeta potential differences between some biomolecules and modified chemical functional groups. By assembling positively charged anti-AFB_1_ antibodies onto the surfaces of a negatively charged Nafion film via electrostatic adsorption, Lin et al. fabricated a competitive-type immunosensing analytical strategy for AFB_1_ detection based on mesoporous carbon nanoparticles [29].

### 2.2. Aptamer-Functionalized CNMs

Aptamers can bind to specific targets with high affinity and specificity by folding into different secondary or tertiary structures [30,31]. Due to their greater stability towards hydrolysis and easier modification than antibodies, aptamers were proven to be a better recognition element than antibodies. Therefore, it is very important to combine the adapter with the electrode surface of the sensor.

**Figure 3:**
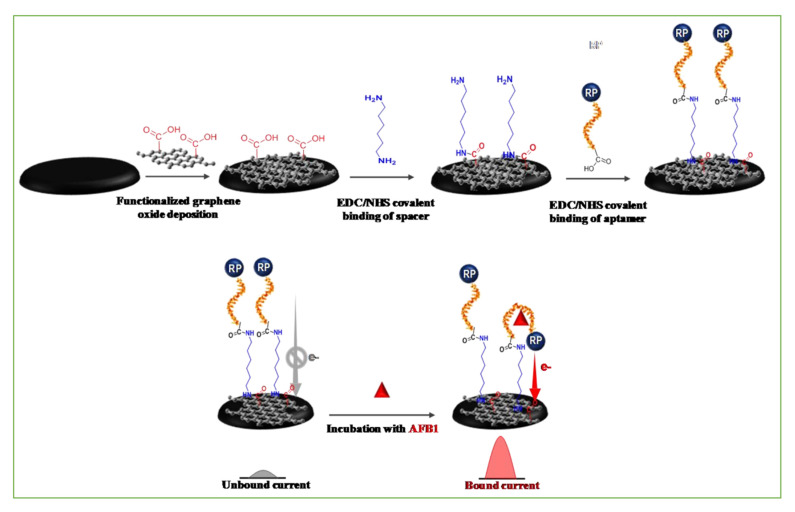
Schematic representation of the electrode fabrication and principle of the developed molecular folding-based aptasensor. Reprinted with permission from [32]. Copyright 2017 Elsevier.

Most of the aptamers were combined with carbon-based electrodes via covalent coupling. The carboxyl terminus on the surface of carbon nanomaterials was activated by EDC and then covalently coupled with the amino group of the 5-amino modified aptamer. Subsequently, the modified aptamer was immobilized onto the surface of the carbon nanomaterials through an amidation reaction (Figure 3) [32]. Combining an aptamer on the surface of a GO-modified electrode in this way, Gonca and colleagues developed an electrochemical aptasensor involving the synergistic effect of GO and a nanoceria (nCe) tag for the detection of OTA in cereal samples [33]. To enhance the binding ability of the adaptor, in addition to modifying the carbon material itself, it can also be compounded with other materials. Au, as an excellent carrier for the immobilization of aptamers, is often composited with carbon materials. The 3D-rGO/Au NPs were synthesized using a one-pot method coated on a glassy carbon electrode, providing a large binding site for the -SH-modified aptamer through unique Au-S connections. Yasmin and colleagues used electrochemical impedance spectroscopy (EIS) for testing and showed a linear response from 1 pg/mL to 10 ng/mL with a limit of detection (LOD) of 0.34 pg/mL for OTA detection in red wine samples [34]. The plentiful presence of carboxyl groups in the CQDs provided strong bonding of aptamers on the surface of CQDs with π-π interactions [35]. Rahimi and colleagues developed an ultrasensitive aptasensor based on an AFB_1_ aptamer immobilized on CQD/octahedral Cu_2_O nanocomposites [36].

In addition to the modification of aptamers or carbon materials, carbon materials and aptamers can also be combined through electrostatic attraction, demonstrating spontaneous self-assembly between molecules. Benefitting from the nucleic acid aptamer labeled with FAM being adsorbed on nanographite through π-π accumulation between the nucleotide base and nanographite, Wei et al. designed an aptasensor utilizing a nanographite-aptamer hybrid and DNase I for the amplified detection of OTA in real red wine samples and achieved the detection of OTA with a limit of 20 nM [37]. Through aptamers spontaneously self-assembling in aqueous solution through the hydrophobic driving force between the DNA base and the SWNT sidewall, Guo et al. constructed a fluorescent aptasensor for the detection of OTA in beer, utilizing SWNTs as quenchers, which can quench the fluorescence of free unfolded aptamers attached to FAM (carboxyfluorescein). The detection limit of the SWNT-based sensor platform without any coating material was 24.1 nM, and the linear detection range was 25 nM to 200 nM [38].

### 2.3. MIPs Decorated CNMs

MIPs can be customized according to the molecular structure of the target to have specific recognition characteristics, which are very suitable for the identification of sensor components [39,40]. MIPs were created through the polymerization of a functional monomer in the presence of an analyte template [41], such as pyrrole, ethyl 3-coumarincarboxylate, p-aminobenzoic acid, etc. Most MIP films were prepared on the surface of the modified electrode by self-assembly and copolymerization of the functional monomer and the template. MIPs are usually deposited on the surface of carbon nanomaterials to detect mycotoxins.

Through a nonhydrolytic sol-gel process, MIP self-assembled on the CD surface in the presence of 1,8-dihydroxyanthraquinone as an alternative template molecule to obtain CDs@MIP. Xu and colleagues designed a sensitive fluorescent sensor for the determination of sterigmatocystin (ST) in grains [42]. In situ electrochemical polymerization has been widely used as a promising surface imprinting method due to its simplicity and quick execution, easy control of polymer film thickness, good electrode surface adhesion, and high reproducibility. Through the electropolymerization of pyrrole on the surface of MWCNTs by cyclic voltammetry (CV), Pacheco and colleagues fabricated a novel electrochemical sensor for OTA detection in spiked beer and wine samples [43]. The synthetic Au@Cu-MOF was then applied to the surface of nitrogen-doped graphene quantum dots (N-GQDs)/GCE and dried to obtain Au@Cu-MOF/N-GQDs/GCE to detect patulin in apple juice. The MIP film was formed by the electropolymerization of aniline as a functional monomer and patulin as a template at Au@Cu-MOF/N-GQDs/GCE. The designed MIP electrochemical sensor showed a wide linear range from 0.001 to 70.0 ng mL^−1^ and a low detection limit (0.0007 ng mL^−1^) [44].

### 2.4. Carbon-Based Nanocomposites

Nanocomposite materials can integrate the advantages of their components, thus significantly improving the detection performance of mycotoxins. Metals, metal oxides, metal sulfides, and others were immobilized on the surface of carbon-based electrodes via adsorption mechanisms and self-assembly. For example, using metal nanoparticles as self-assembled monolayers on the surface of carbon materials as electrodes greatly improves electron transfer. The combination of various metals and metal oxide nanoparticles with graphene derivatives enhances its electrocatalytic performance [45,46]. There are two ways to bind or load metal and metal oxide nanoparticles with graphene: immobilized in situ hybridization and in situ binding or crystallization [47,48,49].

#### 2.4.1. Nanostructured Nobel Metal-Doped CNMs

Since the surface plasmon resonance of noble metal nanostructures (such as Au and Ag) was found to enhance the photoelectric conversion of large-bandgap photoelectric materials in the visible and NIR regions with good stability, it has been widely used in the construction of sensors [50]. Au NP-modified rGO sheets provide a very large electrochemically active surface area, resulting in rapid multiphase electron transfer kinetics and high electrocatalytic activity [51,52]. Usually, the metal is bonded to the surface of GO or CNTs via a simple electrochemical method or in situ thermal reductions of hydrogen tetrachloroaurate(III) (HAuCl_4_). Composites of GQDs-Au NPs have led to great interest in the development of electroanalytical devices due to the combination of interesting properties such as a large surface-to-volume ratio and high catalytic activity. Therefore, the composite of GQDs and Au NPs was synthesized by chemical conjugation and can be used to detect AFB_1_ [53]. Positively charged N,C-dots were synthesized by the hydrothermal treatment of pancreatin. Then, they were assembled on aptamer/Au NPs by the electrostatic interaction-sensitive detection of AFB_1_. The unique plasmon resonance effect of Ag NPs produces a sensitive response. rGO nanosheets serve as substrates for the in situ growth of Ag@AgCl nanoparticles and improve charge separation and transportation. The prepared Ag@AgCl/rGO heterostructure exhibited excellent photocurrent response and stability under visible light irradiation. A highly sensitive photoelectrochemical immunosensor for OTA was constructed [54].

#### 2.4.2. CNMs Support Metal Oxide Nanoparticles

Magnetic Fe_3_O_4_ nanoparticles (Fe_3_O_4_NPs) and rGO were constructed by in situ synthesis. A chemical bond (Fe-O-C) formed between Fe_3_O_4_NPs and unreduced epoxy groups in rGO to combine them tightly, forming a structurally stable Fe_3_O_4_NP/rGO nanocomposite structure. Through the excellent catalytic properties of Fe_3_O_4_NPs, an electrochemical aptasensor for patulin based on tetrahedral DNA and a thionine (Thi)-labeled Fe_3_O_4_NP/rGO signal amplification strategy was designed [55]. Cu_2_O particles were strongly attached to the surface of CQD nanoparticles by the in situ crystallization method. CQD-Cu_2_O nanocomposites were used to improve the immobilization efficiency of the aptamer, thereby improving the electron transfer and increasing the sensitivity of the aptasensor [56]. Therefore, combined with the good electrochemical performance of octahedral Cu_2_O and the water solubility and biocompatibility of CQDs, an electrochemical aptasensor based on a Cu_2_O-CQD nanocomposite for the detection of AFB1 in a wheat flour sample was constructed [36]. ZnO and NGQDs were constructed by a one-step in situ conjugate method. ZnO-NGQD composites were formed via the chemical reaction between ZnO and NGQDs through Zn-O-C bonds during in situ synthesis. As the introduction of NGQDs can efficiently restrain the recombination of charge-hole pairs and improve the photoelectric conversion of ZnO, a photoelectrochemical aptasensor for the detection of ZEN in mildewing cereal crops was constructed [57].

#### 2.4.3. Others

The dispersion capabilities and electronic conductivities of MoS_2_ nanosheets can be significantly improved when MoS_2_ nanosheets are synthesized in situ on rGO templates [58,59,60]. A novel rGO/MoS_2_/polyaniline@Au NP-based electrochemical aptasensor for the detection of AFB_1_ in wine was developed [61]. WS_2_ can be functionalized by MWCNTs to form a WS_2_/MWCNT nanocomposite structure. The bare GCE was modified with WS_2_/MWCNTs by drop-casting. The negative charges on the WS_2_ surface tended to reduce the aggregation and restacking of MWCNTs. Through the perfect conductivity of WS_2_/MWCNT nanomaterials, a voltammetric immunosensor was described to realize the rapid and ultrasensitive detection of AFB_1_ by WS_2_/MWCNTs [28]. Carbon nanomaterials are functionalized with different nanomaterials (as shown in Table 1).

Although these materials have excellent properties, due to the low solubility and hydrophobic interaction of graphene and CNTs in an aqueous solution, they easily agglomerate, thus limiting their use. The common solution is to use carbon material to compound on the electrode surface while adding chitosan for dispersion. Functionalization can also solve this problem. Covalent immobilization methods often depend on amide bonds formed between CNMs and antibodies/aptamers. Since covalent binding is very stable, it is desirable for high-sensitivity biosensing. Noncovalent binding is another way to bind CNMs and antibodies/aptamers for biosensing. The noncovalent immobilization methods mainly depend on electrostatic forces, hydrogen bonding, and π-π interactions. These methods are easy to operate and do not affect the intrinsic structure and original properties of MOFs. Therefore, multiple considerations should be taken into account in the preparation process of sensors, and appropriate functionalization methods should be selected.

## 3. CNM-Based Smart Sensor for the Detection of Mycotoxins

Compared with simple sensors, molecular recognition element-based sensors offer highly sensitive detection with enhanced selectivity towards mycotoxins. Biorecognition units, such as antibodies, aptamers, and MIPs, have high specificity to detect mycotoxins. In addition, nanomaterials can not only improve the signal detection ability but also improve the ability to combine with the biorecognition unit. Thus, the combination of biorecognition units with functional carbon nanomaterials may greatly improve the detection effect.

### 3.1. Smart Sensors Based on Antibodies

Immunosensors are commonly applied analytical tools that adopt antibodies as the recognition element and a transducer, which in turn translates the antigen-antibody binding event to a measurable physical signal [63,64]. At the same time, they are generally simple to operate and can easily realize digitization, automation, and miniaturization [65,66]. Over the last few years, rapid, inexpensive, simple, and sensitive carbon-based immunosensors for mycotoxin detection have received increasing attention.

**Figure 4:**
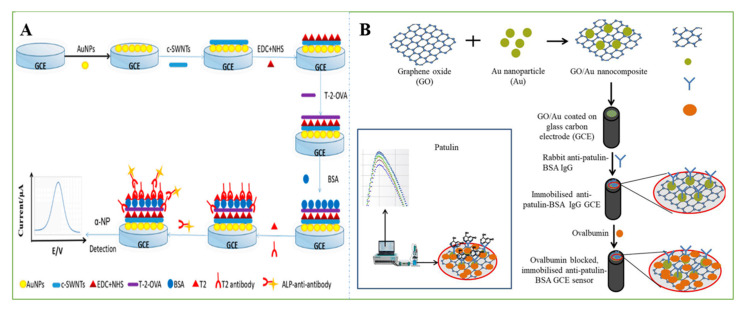
(**A**) The fabrication process of this electrochemical biosensor [62]. Reprinted from MDPI, 2018. (**B**) Schematic illustration on the preparation of immobilized GCE sensor for the detection of patulin. Reprinted with permission from [69]. Copyright 2021 Elsevier.

The good electrical conductivity, high surface-to-volume ratio, rapid electrode kinetics, and biocompatible nature of SWCNTs have been improved by immobilizing SWCNTs with various biomolecules (such as antibodies) for various sensing applications. For example, Abera et al. [67] developed an EC method for the ultralevel identification of aflatoxin M_1_ in milk. Due to the large surface area of the SWCNTs, a large number of antibodies were covalently attached to the surfaces of the SWCNTs, suggesting that the SWCNTs act as supports for the antibodies. Flexible biosensors were fabricated using dispense-printed electrodes, which were functionalized with SWCNTs and subsequently coated with specific antibodies to improve their sensitivity. The functionalized SWCNTs were used as electrodes for AFM_1_ detection. The proposed EC sensor offered a lower LOD of 0.02 µg/L with high selectivity. Zhang et al. designed an indirect competitive AFB_1_ electrochemical immunoassay based on SWCNTs/chitosan. The mechanism of the EC immunosensor was indirect competitive binding to a certain amount of anti-AFB_1_ between free AFB_1_ and AFB_1_-bovine serum albumin. Then, alkaline phosphatase, a labelled anti-mouse secondary antibody, catalyzed the hydrolysis of the substrate α-naphthyl phosphate, which produced an electrochemical signal to assay AFB_1_ in corn powder. Differential pulse voltammetry (DPV) test results showed that the current density decreased linearly with the logarithm of the AFB_1_ concentration between 0.01 and 100 ng/mL, and the detection limit was as low as 3.5 pg/mL [26]. Similarly, the team also used the same principle to design electrochemical sensors to detect FB_1_. To realize multiple signal amplification, gold nanoparticles were combined on the surface of the electrode, and a specific EC immunosensor based on an Au NP/cSWNT/chitosan composite was established using the same principle. Under optimal conditions, this method could quantitatively detect T-2 in swine meat from 0.01 to 100 µg L^−1^ with a detection limit of 0.13 µg L^−1^ (Figure 4A) [62].

Apart from CNTs, graphene-coated electrodes can also serve as efficient EC sensors with better performance than bare electrodes [68]. To further enhance the EC properties of sensors, graphene nanocomposites have been coated with various molecules and successfully integrated with EC devices for the detection of mycotoxins with both high selectivity and high sensitivity. Through the layer-by-layer electrochemical deposition method, a simple and rapid electrochemical AFB_1_ sensor based on Au NP/graphene nanosheets that could enhance the Raman effect and electrochemical conductivity was developed. AFB_1_ was monitored by recording changes in the redox current response and Raman spectroscopy of the AF-antibody adsorbed on Au/graphene/ITO. Raman spectra and CV techniques were used to monitor the presence and quantitative detection of AFB_1_. It showed a low LOD of approximately 6.9 pg/mL in spiked peanut aliquot samples [27]. In another study, a GCE was modified with anti-patulin-BSA IgG/GO/Au NPs for the EC immunosensing of patulin in food. Since the spatial hindrance effect of IgG on the GCE sensor was reduced by the reaction between IgG and patulin, the electron transfer resistance was decreased. Due to the cross-reaction with the carrier protein BSA, this sensor detected patulin at concentrations as low as 5 µg/L in less than 1 min without the presence of BSA (Figure 4B) [69].

To optimize the characteristic properties of graphene, the transformation scheme of the nanostructure material of 2D graphene sheets to OD quantum dots was implemented. Numerous properties have been improved, such as electronic and optical stability and robust chemical inertness. For example, an EC label-free immunosensor based on GQDs/Au NPs/ITO was described for the detection of AFB_1_ in maize samples. Electrochemical signals via antigen-antibody interactions were investigated using EIS and CV techniques in a hexacyanoferrate redox probe. The linear range of AFB_1_ was 0.1–2.5 ng/mL, and the detection limit was 0.11 ng/mL [53]. AFB_1_ is highly specific and has good reproducibility and acceptable stability. The method can also be exploited to sense other mycotoxins by using their respective antibodies. In another study, GQDs were decorated on MoS_2_ nanosheets as an active electrode material to enhance the electrochemical performance of the analyte detection system. A label-free electrochemical ultrasensitive biosensor was fabricated for AFB_1_ detection using MoS_2_@GQDs as an electrode surface transducer material. The immunosensor via the electrochemical method showed a signal response in the AFB_1_ concentration range of 0.1 to 3.0 ng/mL in a spiked maize sample with a detection limit of 0.09 ng/mL [70].

Despite the high sensitivity and specificity of analyte identification, the antibodies described showed some limitations, namely, the need for animal production at least in the first stage, variability and hydrolytic degradation, and incompatibility with many organic solvents required for mycotoxin extraction. In addition to fragmented natural antibodies, immunoglobulins consisting of only heavy chains (nanobodies) have received increasing attention, showing excellent solubility, elevated stability, high affinity, and good specificity. These monomeric antibody fragments can not only be easily genetically manipulated and expressed but also have high yields and low cost, which will prolong the shelf-life of the immunosensors due to their stability.

### 3.2. Smart Sensors Based on Aptamers

To date, aptamers with specificity and selectivity for AFB_1_, OTA, FB_1_, and ZON have been screened [71,72,73,74]. With the addition of aptamers, the detection performance of nanosensors has been greatly improved. They will also enhance the conductive and catalytic properties of the sensor and modulate the interface morphology, thereby enhancing ligand-aptamer interactions. Usually, we use optical and electrochemical aptamer-based assays for mycotoxins [75]. To achieve the cost-effective and high sensitivity detection of mycotoxins, various nanomaterial-based aptasensors were developed (as shown in Table 2).

#### 3.2.1. Optical Aptasensors

An optical sensor uses a biorecognition unit and a specific combination of the measured object, and the reaction can produce the output of the optical signal through the detection of the change in the optical signal to achieve qualitative or quantitative detection of the target sensor. The combination of aptamers with carbon nanomaterials with fluorescence or fluorescence quenching properties can be well used for the detection of mycotoxins.

The fluorescence quenching effect of water-soluble carbon materials, including GO, CNHs, and CNTs [77,101,102,103,104], has been successfully applied to the development of fluorescence biosensor platforms based on aptamers. For example, due to the π-π stacking effect between the nucleobases of aptamers and sp^2^ atoms of GO, UCNP-labelled aptamer sequences can be tightly adsorbed onto the GO layer. In fluorescence resonance energy transfer (FRET), energy transfers between upconversion fluorescent nanoparticle (UCNP) donors and GO acceptors. In the absence of the mycotoxins OTA and FB_1_, the fluorescence values of the aptamer-modified UCNPs were decreased due to the strong quenching effect of GO [74].

**Figure 5:**
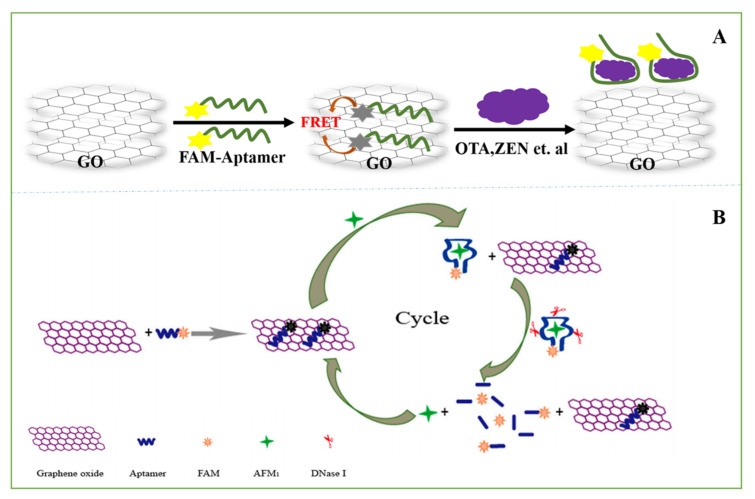
(**A**) Schematic diagram of an aptamer for detection of mycotoxins based on FRET. (**B**) Illustration of the aptasensor for the detection of aflatoxinM1(AFM1). FAM: carboxyfluorescein [115]. Reprinted from MDPI, 2019.

When the fluorescence spectrum of one fluorescent molecule (also known as the donor molecule) overlaps with the excitation spectrum of another fluorescent molecule (also known as the recipient molecule), the excitation of the donor fluorescent molecule can induce the recipient molecule to emit fluorescence, and the fluorescence intensity of the donor fluorescent molecule itself decays. Mycotoxin optical sensors of carbon-based nanomaterials are mostly designed based on the principle of FRET (Figure 5A).

Exfoliated functional graphene oxide (FGO) with high water dispersibility was adopted as an effective fluorescence quencher of the fluorescence of FAM. A rapid FRET-sensing platform was constructed for the highly sensitive and selective detection of ZEN. The LOD value was 0.5 ng·mL^−1^ with a linear calibration plot in the range of 0.5 to 64 ng·mL^−1^ ZEN in alcoholic beverage samples, beer, and wine [83]. To achieve a simple, sensitive and turn-on sensing method for the target molecules, several fluorescent aptasensors were developed by synthesizing a series of aggregation-induced emission (AIE) molecules (9,10-distyrylanthracene derivatives, DSAs) as fluorescent probes [105,106,107,108,109,110,111]. Ultrasensitive FRET-based detection of OTA was achieved using a hybrid matrix composed of GO modified with DSAI and DNA (DSAI-ssDNA-GO). The aptamer 5′-GAT CGG GTG TGG GTG GCG TAA AGG GAG CAT CGG ACA-3′ was adsorbed on the surface of GO due to the strong π-π stacking interactions, which was labeled by DSAI. By introducing GO and DSAI, the fluorescence signal of DSAI can be easily turned from “off” to “on” after the addition of OTA. The LOD value was 0.324 n mol/L with a linear calibration plot in the range of 10–200 nmol/L OTA in red wine samples [78].

The addition of exonuclease can release the target substance that has been bound by the aptamer into the solution and enter the next detection cycle, thus playing the role of expanding the signal and improving the detection sensitivity. ssDNA can interact with GO through π stacking between DNA bases and hexagonal cells of GO. After noncovalent adsorption onto the GO surface, single-stranded DNA (ssDNA) and RNA were effectively protected from enzymatic digestion by nuclease due to the steric hindrance effect of GO that prevents nuclease from binding to DNA and RNA [112,113,114]. A rapid and sensitive fluorescent aptasensor for the detection of AFM_1_ in milk powder was developed. With the addition of AFM_1_, the formation of the AFM_1_/aptamer complex causes the aptamer to depart from the surface of GO, and then, the aptamer is cleaved by DNase I and releases the target AFM_1_ into a new cycle, which results in high sensitivity and great signal amplification. This fluorescent aptamer sensor detected AFM_1_ levels in a dynamic range from 0.2 to 10 g/kg, with a LOD of 0.05 g/kg (Figure 5B) [115]. Exonuclease III (Exo III) is a DNA-modifying enzyme that is also widely used in molecular biology. Wu et al. established a fluorescence sensing platform for OTA detection using Exo III-aided signal amplification and the fluorescence quenching function of SWCNHs. The detection scheme employs a hairpin probe (HP), and a signal probe (SP) labelled with carboxyfluorescein (FAM) at its 5′-end. The linear range of this method was from 10 nM to 1000 nM, and the limit of detection was 4.2 nM. By adding a fluorescent agent to the 5′-end of the aptamer, the sensor detects the labeled OTA of beer and wine and finds it to be free of interference from the sample matrix [77].

**Figure 6:**
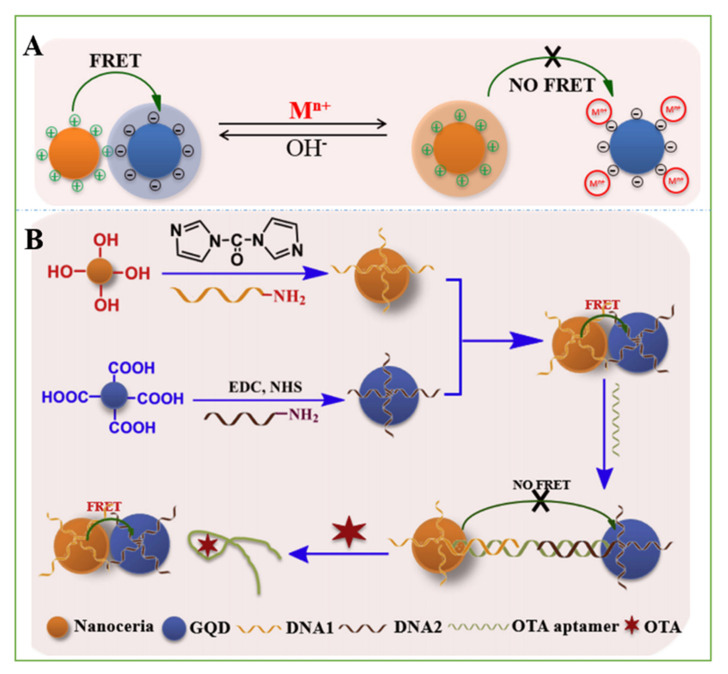
(**A**) Schematic process of the effect of metal cation on FRET between nanoceria and GQD under different conditions. (**B**) Schematic process of FRET aptasensor for OTA determination. Reprinted with permission from [82]. Copyright 2018 Elsevier.

In addition to fluorescence quenching, some carbon nanomaterials have fluorescent properties, which can be used to detect mycotoxins when they are combined with aptamers. Nitrogen-doped C-dots (N,C-dots) possess particularly rich photophysical properties due to the presence of nitrogen-containing functional groups on the surfaces of the C-dots [116,117]. A sensitive AFB_1_ sensor based on N,C-dots/aptamer/Au NPs was fabricated. N,C-dots were assembled on the aptamer/Au NPs by electrostatic interaction, resulting in effective fluorescence quenching of N,C-dots. When AFB_1_ was added to the assay solution, specific interactions between the aptamer and AFB_1_ caused the release of N,C-dots. The N,C-dot fluorescence recovery rate can be used for the quantification of AFB_1_. The detection limit of this method was 5 pg/mL (16 pM), and the linear range was 5 pg/mL to 2.00 ng/mL [118]. An excellent donor-acceptor pair is an important factor in improving the efficiency of ratiometric FRET and the analytical performance. Tian et al. established a sensitive and selective aptasensor for the detection of OTA in peanuts. By adsorbing each other and leading to the occurrence of FRET, probes DNA1@nanoceria and DNA2@GQD were designed to complement the OTA aptamer. After adding the OTA aptamer and then introducing OTA, FRET was interrupted/recovered due to the specific affinity of OTA and its aptamer, and the fluorescence recovery value increased with the addition of OTA. The sensor showed good accuracy; the linear response range was 0.01–20 ng mL^−1^ OTA, and the LOD was 2.5 pg mL^−1^ (Figure 6) [79].

A fluorometric and aptamer-based assay using humic acid as a florescent quenching agent for AFB_1_ in peanut oil was described. Due to its rich structure, including abundant quinoid units and aromatic rings, humic acid has a strong affinity for ssDNA [119] through π-π stacking interactions. In this work, blue fluorescent CDs were combined with nucleic acid aptamers as fluorescent probes (DNA-CDs). Then, the DNA-CD fluorescent probe was reacted with HAs, and its fluorescence was quenched. If the nanoprobe reacted with AFB_1_, the DNA-CDs detached from the HAs, and fluorescence was restored. The linear range of this experiment was 0.1–0.8 ng mL^−1^, and the low limit of detection was 70 pg mL^−1^ [120].

#### 3.2.2. Electrochemical Aptasensors

Biosensors based on electrochemical measurements usually depend on the investigation of active reactions that generate a measurable current, potential change, or impedance generated from conductance change, and these changes can be read by CV, DPV, EIS, photoelectrochemical (PEC), and electrochemiluminescence (ECL) analysis, respectively [121]. The integration of carbon nanomaterials into the structure of EC aptasensors offers great advantages in mycotoxin detection, which has resulted in unprecedented success in mycotoxin analysis and monitoring.

The performance of EC aptasensors can be clearly improved when carbon nanomaterials are used to fabricate EC transducer systems of aptasensors. A nonenzymatic nanocatalyst-based competitive EC aptasensor was fabricated for the sensitive detection of OTA in cereal samples by the synergistic contribution of a nanoceria (nCe) tag and GO. The SPCE was coated by carboxyl GO, which not only promoted electron transfer between the SPCE surface and the catalytic label to amplify the EC signal but also acted as an efficient substrate for increasing the loading of many aptamers. Due to the presence of cerium in Ce^3+^/Ce^4+^ dual oxidation states, nCe acts as a redox catalyst for the amplification of the EC oxidation of H_2_O_2_ [122]. The high conductivity and peroxidase-like activity of carboxyl GO can further enhance the catalytic conversion of H_2_O_2_ by nCe. In the presence of OTA, the EC signal of H_2_O_2_ was decreased because OTA can bind with an immobilized aptamer via a competitive mechanism involving nCe-labeled OTA and free OTA. OTA was detected by monitoring the electrochemical signals of CV and EIS generated by the electrooxidation of common REDOX substances when they react with nCe tags. This aptasensor exhibited a linear response in the range of 0.15−180 nM with a detection limit of 0.1 nM [33].

**Figure 7:**
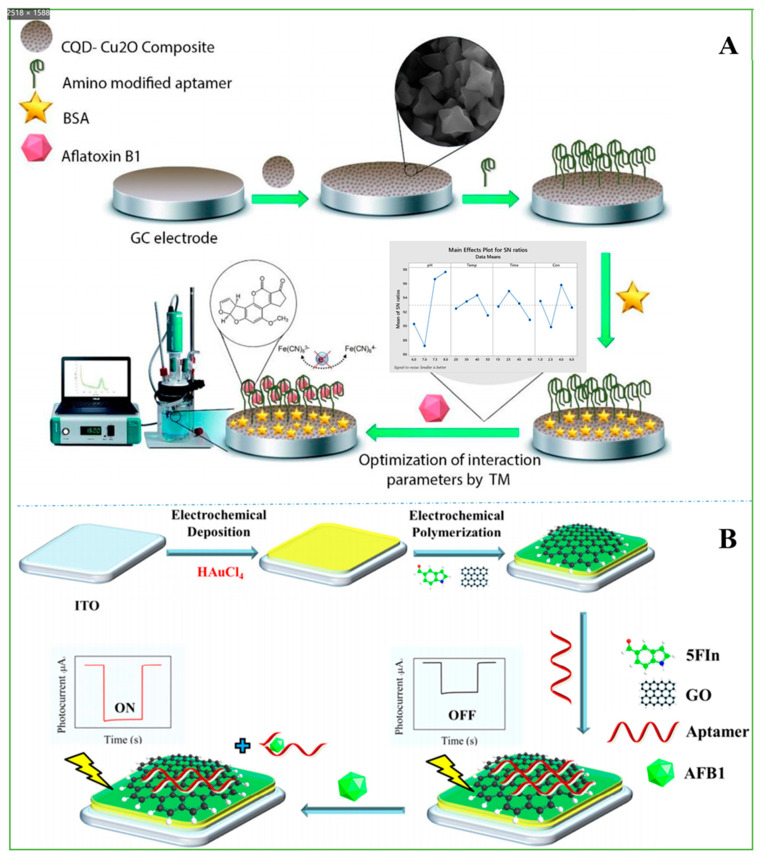
(**A**) Schematic of construction and performance stages of the proposed aptasensor. Reprinted with permission from [36]. Copyright 2021 Elsevier. (**B**) Schematic illustration of PEC aptasensor. Reprinted with permission from [90]. Copyright 2019 Elsevier.

A sensitive label-free aptasensor assembled with rGO nanosheets as the signal amplifier was fabricated for detecting ultralow levels of AFB_1_ in pasteurized cow milk and human blood plasma as real samples. A “sandwich” structure in which the aptamer was fixed between the surface of the electrode and rGO was used. The rGO nanosheets can bind to the aptamer on the other side of the assembly through π-π interactions, which significantly reduces the charge transfer resistance associated with the REDOX process. When AFB_1_ was present in the solution, the aptamer folded and discarded the rGO nanosheets. The AFB_1_ content was analyzed by the change in REDOX of the electrochemical probe K_4_[Fe(CN)_6_]/K_3_[Fe(CN)_6_] in the solution. The biosensor detected AFB_1_ in a wide linear range (0.5 nM-4 µM) by DPV with a low limit of detection (LOD = 0.07 nM) [87]. To further improve the detection performance of the sensor. The metal nanoparticles used to fabricate the sensor show high electrocatalytic activity in the detection of analytes [123]. An ultrasensitive aptasensor was fabricated based on an AFB_1_ aptamer immobilized on a CQD/Cu_2_O nanocomposite. The GCE was coated with the CQD-Cu_2_O nanocomposite, which not only increased the immobilization efficiency of aptamers but also increased the immobilization efficiency, causing more electron transfer and increasing the aptasensor sensitivity. In this study, electrochemical measurements were based on EIS and DPV. The AFB_1_ dynamic range of 3 ag ml^−1^−1.9 µg ml^−1^ and a low LOD of 0.9 ± 0.04 ag ml^−1^ were detected (Figure 7A) [36].

In addition, a “signal-on” PEC aptasensor was constructed for AFB_1_ detection in real peanut and wheat samples based on electrochemical rGO/poly(5-formylindole)/Au (erGO/P5FIn/Au) nanocomposites with a strong photocurrent response. After the AFB_1_ aptamer was immobilized on the erGO electrode, the PEC sensor signal was “OFF”. When AFB_1_ combined with the aptamer, the aptamer detached from the surface of erGO, which resulted in the sensor signal being “ON”. AFB_1_ was detected with a wide linear detection range (LDR) from 0.01 ng mL^−1^ to 100 ng mL^−1^ and a low LOD of 0.002 ng mL^−1^ (Figure 7B) [90].

Aptamers have aroused much attention from researchers as an alternative to antibodies since they are more flexible, cost-effective, and stable because they are more stable than conventional immunoglobulins and easily adapted to various applications. However, due to their high sensitivity, they are also affected by environmental variables such as the salt concentration and pH value, and different biosensors need to be optimized separately, increasing the time and complexity of development.

### 3.3. Smart Sensors Based on MIPs

MIPs recognize targets with patterns similar to those of antibody-antigen and receptor-ligand interactions, but the latter biomaterial’s poor stability and harsh conditions limit its application. Nanomaterials are characterized by strong adsorptivity and diffusivity and extremely high surface reactivity and catalytic activity [124,125]. When MIPs are combined with carbon nanomaterials, they can effectively improve the detection of mycotoxins.

An electrochemical sensor for OTA detection was fabricated through the decoration of a GCE with MWCNTs and a MIP. MWCNTs were used to increase the surface area and conductivity of the sensor. The imprinted polypyrrole film was prepared by electropolymerization of pyrrole in the presence of OTA as a template molecule via CV. Then, the MIP/MWCNT/GCE was eluted to completely remove the OTA molecules, creating specific binding cavities. The sensor was used for the detection of beer and wine samples. OTA was detected with DPV with a linear range between 0.050 and 1.0 μM and a limit of detection of 0.0041 μM [43].

**Figure 8:**
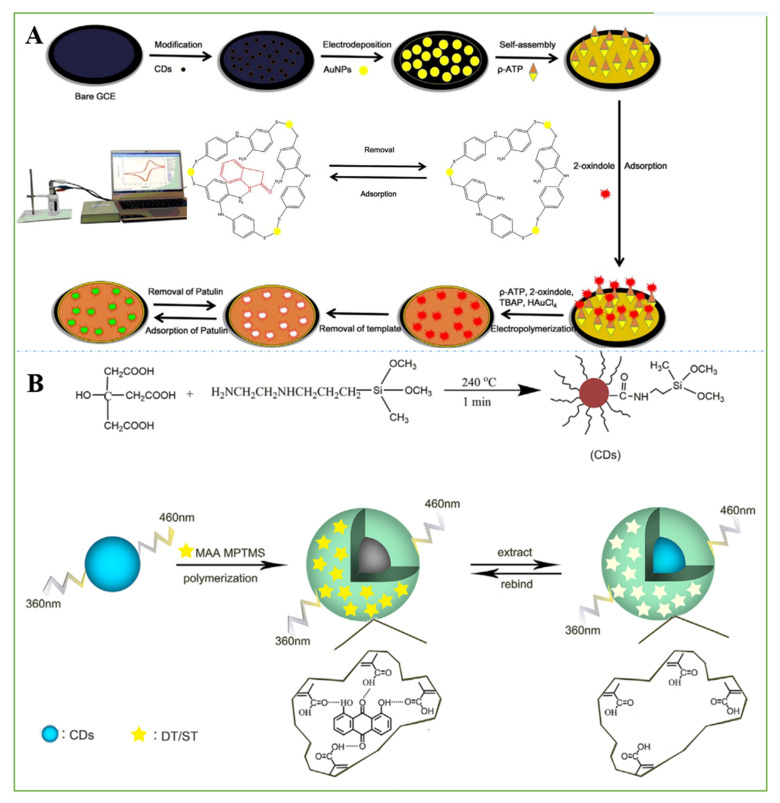
(**A**) Preparation procedure of the MIP-Au/CS-CDS/GCE. Reprinted with permission from [127]. Copyright 2017 Elsevier. (**B**) Preparation of CDs@MIP. Reprinted with permission from [42]. Copyright 2016 Elsevier.

To enhance the signal response, CdS quantum dots were combined with an appropriate amount of GO to form a heterojunction. An original solution of MIP was deposited on the surface of the electrode by ultraviolet photopolymerization. When the MIP sensor was eluted in ethanol, its photocurrent response was significantly restored because the template molecules were washed away, and electron donors entered the holes and accelerated the electron transfer. Its photocurrent response was reduced because holes were blocked when the MIP-PEC sensor was hatched in the template molecule culture fluid. It has a linear range from 0.01 to 1000 ng mL^−1^ with a detection limit of 4.7 pg mL^−1^ for FB_1_ in real samples [126]. CDs and chitosan can improve the electron transfer rate, expand the electroactive surface of the electrode, enhance the signal strength, and have other advantages as surface modification materials for GCEs. The MIP electrochemical sensor senses patulin in fruit juice by changes in electrical signals. The linear response range for the detection of patulin was from 1 × 10^–12^ to 1 × 10^−9^ mol L^−1,^ and the LOD was 7.57 × 10^–13^ mol L^−1^ (Figure 8A) [127].

In addition to electrochemical detection, it was reported that the high selectivity of molecularly imprinted polymers and the stable fluorescence characteristics of CDs have been used to realize the sensitive and selective detection of ST. In this study, CDs were wrapped in the MIP, 1,8-dihydroxyan-thraquinone, as a selective recognition, signal amplification, and optical readout element, which serves as an alternative template to provide specific binding sites for ST. In the presence of ST, the fluorescence of CDs@MIP was quenched, and the fluorescence quenching process was proportional to the concentration of ST in the sample. The sensor was also applied to the determination of the ST content in grain with satisfactory results. There was a linear range from 0.05 to 2.0 mg L^−1^ with a detection limit of 0.019 mg mL^−1^ for ST (Figure 8B) [42].

At present, there are still some problems with MIP sensors: (1) it has been reported that the preparation process of various types of MIP sensors is cumbersome, and the response time is too long, and (2) most molecularly imprinted polymers can only be polymerized and applied in organic phases, while most natural molecular recognition systems are carried out in aqueous solutions. How to carry out molecularly imprinted polymers and recognition in aqueous solutions or polar solvents is still a major problem. The ideal MIP should have the following properties: (1) it should be rigid enough that the polymer can retain the original shape and size of the hole after removing the template molecule; (2) it should be flexible enough that the binding between the substrate and the hole can reach a balance quickly; (3) the imprinted sites on the MIP should be accessible; (4) it has certain mechanical stability; and (5) it has thermal stability thus that it can be used at higher temperatures.

### 3.4. Others

In addition to the above-modified biorecognition unit, nanomaterials are also combined with others to detect mycotoxins. For example, a rGO/SnO_2_ composite for the electrochemical detection of PAT that does not require a biological or chemical receptor or specific antibodies was synthesized, which showed outstanding performance and demonstrated promising electrochemical properties in the direct detection of PAT levels in contaminated apple juice samples. The DPV response of the rGO/SnO_2_ composite electrode via the changes in electrical signals generated by the reduction of PAT by SnO_2_ showed a linear relationship with the PAT concentration in the 50–600 nM range and had a lower detection limit of 0.6635 nM [128]. In another study, an amperometric sensor based on a step-by-step modification of the bare GCE by graphene-multiwalled carbon nanotube-chitosan-ionic liquid (Gr-MWCNTs-Ch-IL)/collagen-IL (CG-IL)/NiO NPs for the ultrasensitive determination of OTA in juice samples was fabricated. The sensor was able to ultrasensitively determine OTA in a concentration range of 0.01 nM to 10 nM with a limit of detection of 0.5 × 10^−11^ M and a sensitivity of 36.4 μA nM^−1^ [129].

## 4. Conclusions and Future Perspectives

This review was organized to describe the integration of biorecognition units (antibodies, aptamers, and MIPs) and CNM/carbon-based nanocomposites in smart sensors for the ultratrace identification and quantification of mycotoxins in various samples. As presented in the literature, smart sensors have been regarded as powerful testing devices due to their numerous advantages, such as miniaturization, high sensitivity and selectivity, low cost, simple design, and shortened analysis time. The analytical efficiency of smart sensors has improved tremendously through the modification of electrodes with biorecognition unit functionalized carbon-based nanomaterials. Therefore, based on the research status of mycotoxins in food products, this review highlights the recent advances of various newly developed smart sensors for mycotoxin analysis, with a particular emphasis on electrochemical sensors and optical sensors and further discusses their advantages and potential limits as well as future perspectives.

Although the developed smart sensors have displayed excellent performance and an encouraging future in mycotoxin detection, they still face various challenges that need to be solved. Researchers have made unremitting efforts to develop smart sensors for detecting mycotoxins. Since they are only laboratory validated, these sensors are still not mass-produced for end users. Therefore, future studies on fabricating smart sensors for mycotoxin detection could concentrate on the following issues: (1) synthesizing novel nanomaterials with low cost, eco-friendliness, a large surface area, and high adsorption capacity, and great recyclability for their wide applications in smart sensors; (2) developing new biorecognition units for more selective, sensitive and general detection; (3) sensing schemes for label-free multimycotoxin analysis; (4) combination with smartphones or portable devices to realize real-time monitoring and continuous detection; and (5) to achieve multiple continuous uses with no residual dirt on the surface of the smart sensor. Thus, we expect future research on smart sensors to show a significant impact in realizing practical portable devices for the detection of multiple mycotoxins in food products. The device can be applied to food quality control and the food processing and manufacturing industry.

## Figures and Tables

**Table 1:** Carbon nanomaterial composites for mycotoxin detection.

Materials	Method	Mycotoxin	Samples	Linear Range	LOD	Ref.
Au nanodots/rGO	layer-by-layer electrochemical deposition	AFB1	peanut	up to 300 ppb	6.9 pg/mL	[27]
MWCNTs/WS2	drop-casting	AFB1	corn	0.5 to 10 ng mL^−1^	68 fg mL^−1^	[28]
rGO/Au	one-pot hydrothermal	OTA	red wine	1 pg/mL^−10^ ng/mL	0.34 pg/mL	[34]
GQDs/AuNOs	chemical conjugation	AFB1	maize	0.1 to 2.5 ng mL^−1^	0.11 ng mL^−1^	[53]
N-GQDs/Au@Cu-MOF	electropolymerization	patulin	apple juice	0.001 to 70.0 ng mL^−1^	0.0007 ng mL^−1^	[44]
rGO/Ag@AgCl	in-situ synthesis	OTA	red wine	0.05 to 300 nM	0.01 nM	[54]
rGO/Fe_3_O_4_NPs	in-situ synthesis	patulin	apple juice	5 × 10^−8^ to 0.5 μg mL^−1^	30.4 fg mL^−1^	[55]
CQDs-Cu_2_O	in-situ crystallization	AFB1	wheat flour	3 ag mL^−1^–1.9 µg mL^−1^	0.9 ± 0.04 ag ml^−1^	[36]
NGQDs/ZnO	in-situ synthesis	ZEN	cereal crops	1.0 × 10^−13^–1.0 × 10^−7^ g mL^−1^	3.3 × 10^−14^ g mL^−1^	[57]
rGO/MoS_2_	in-situ synthesis	AFB_1_	wine	0.01 fg mL^−1^ to 1.0 fg mL^−1^	0.002 fg mL^−1^	[61]
CSWNTs/Au NPs	electrodeposition	T2	swine meat	0.01 to 100 µg L^−1^	0.13 µg L^−1^	[62]

**Table 2:** Nanocomposite-based aptasensors for mycotoxins.

Mycotoxin	Aptamer Sequence (5′-3′)	Materials	Method	Linear Range	LOD	Ref.
PAT	GGCCCGCCAACCCGCATCATCTACACTGATATTTTACCTT	GO	colorimetric	50–2500 pg mL^−1^	48 pg mL^−1^	[76]
OTA	GATCGGGTGTGGGTGGCGTAAAGGGAGCATCGGACA	SWCNTs	fluorescence	25–200 nM	24.1 nM	[38]
OTA	TCCCTTTACGCCTTTTTGATCGGGTGTGGGTGGCGTAAAGGGAGCATCGGACA	SWCNHs	fluorescence	10–1000 nM	4.2 nM	[77]
OTA	GATCGGGTGTGGGTGGCGTAAAGGGAGCATCGGACA	Nano-graphite	fluorescence	2.0–50.0 μM	20 nM	[37]
OTA	GATCGGGTGTGGGTGGCGTAAAGGGAGCATCGGACA	GO	fluorescence	10–200 nM	0.324 nM	[78]
OTA	GATCGGGTGTGGGTGGCGTAAAGGGAGCATCGGACA	GQDs	fluorescence	0.01–20 ng mL^−1^	2.5 pg mL^−1^	[79]
OTA	GATCGGGTGTGGGTGGCGTAAAGGGAGCATCGGACA	GO	fluorescence	0.08–200 ng mL^−1^	0.08 ng mL^−1^	[80]
OTA	GATCGGGTGTGGGTGGCGTAAAGGGAGCATCGG	GO	FRET	0.05–100 ng mL^−1^	20 pg mL^−1^	[74]
FB_1_	ATACCAGCTTATTCAATTAATCGCATTACCTTATACCAG CTTATTCAATTACGTCTGCACATACCAGCTTATTCAATT AGATAGTAAGTGCAATCT	GO	FRET	0.1–500 ng mL^−1^	100 pg mL^−1^	[74]
AFB_1_	AAAAAAAAAAGTTGGGCACGTGTTGTCTCTCTGTGTCTCGTGCCCTTCGCTAGGCCCACA	GO	fluorescence	up to 300 ppb	4.5 ng mL^−1^	[81]
AFB_1_	GTTGGGCACGTGTTGTCTCTCTGTGTCTCGTGCCCT TCGCTAGGCCC	MWCNTs	fluorescence	0.5–15 ng mL^−1^	20 pg mL^−1^	[82]
ZON	AGCAGCACAGAGGTCAGATGTCATCTATCTATGGTACATTACTATCTGTAATGTGATATGCCTATGCGTGCTACCGTGAA	fGO	fluorescence	0.5–64 ng mL^−1^	0.5 ng mL^−1^	[83]
PAT	GGCCCGCCAACCCGCATCATCTACACTGATATTTTACCT	rGO-Fe_3_O_4_	fluorescence	0.5–30 ng mL^−1^	0.28 ng mL^−1^	[84]
OTA	GATCGGGTGTGGGTGGCGTAAAGGGAGCATCGGACA	GO	Luminescence	0.001–250 ng mL^−1^	1 pg mL^−1^	[85]
OTA	ATCCGTCACACCTGCTCTGACGCTGGGGTCGACCCGGAG AAATGCATTCCCCTGTGGTGTTGGCTCCCGTAT	GO-L-Ag NPs	ECL	10–200 ng mL^−1^	0.05ng mL^−1^	[86]
OTA	GATCGGGTGTGGGTGGCGTAAAGGGAGCATCGGACA	GO	CV	0.15–180 nM	1000 pM	[34]
AFB_1_	GTTGGGCACGTCTTGTCTCTCTGTGTCTCGTGCCCTTCGCTACGCCCACA	rGO	DPV	0.5 nM–4 μM	0.07 nM	[87]
AFB_1_	TGGGGTTTTGGTGGCGGGTGGTGTACGGGCGAGGG	FGO	DPV	0.05–6.0 ng mL^−1^	0.05 ng mL^−1^	[32]
OTA	GATCGGGTGTGGGTGGCGTAAAGGGAGCATCGGACA	SWCNTs	DPV	0–45 nM	58 pM	[88]
OTA	GATCGGGTGTGGGTGGCGTAAAGGGAGCATCGGACA	GONPs	DPV	310 fM–310 pM	310 fM	[89]
AFM_1_	ATCCGTCACACCTGCTCTGACGCTGGGGTCGACCCGGAG AAATGCATTCCCCTGTGGTGTTGGCTCCCGTAT	GO-L-Ag NPs	ECL	5–150 ng mL^−1^	10 pg mL^−1^	[86]
AFB_1_	GTTGGGCACGTGTTGTCTCTCTGTGTCTCGTGCCCT TCGCTAGGCCCACA	erGO	PEC	10 pg mL^−1^–100 ng mL^−1^	2 pg mL^−1^	[90]
T2	GTATATCAAGCATCGCGTGTTTACACATGCGAGAGGTGAA	rGO	Chronoamperometry	10 fg mL^−1^–100 ng mL^−1^	1.79 fg mL^−1^	[91]
FB_1_	ATACCAGCTTATTCAATTAATCGCATTACCTTATACCAGCTTATTCAATTACGTCTGCACATACCAGCTTATTCAATTAGATAGTAAGTGCAATCT	GSTH	CV	1–10^−6^ pg mL^−1^	1 pg m L^−1^	[92]
ZEN	TCATCTATCTATGGTACATTACTATCTGTAATGTGATATG	rGO	DPV	0.5 pg mL^−1^–50 ng mL^−1^	0.105 pg mL^−1^	[93]
ZEN	TCATCTATCTATGGTACATTACTATCTGTAATGTGATATG	MWCNTs	CV	0.5 pg mL^−1^–50 ng mL^−1^	0.17 pg mL^−1^	[94]
OTA	GATCGGGTGTGGGTGGCGTAAAGGGAGCATCGGACA	Graphene	DPV	0.01–1000 × 10^−6^ ng mL^−1^	1 × 10^−7^ ng mL^−1^	[95]
OTA	GATCGGGTGTGGGTGGCGTAAAGGGAGCATCGGACA	Carboxylated graphene	DPV	10 fmol L^−1^–10 nmol L^−1^	3.3 fmol L^−1^	[96]
OTA	GATCGGGTGTGGGTGGCGTAAAGGGAGCATCGGACA	GO	DPV	0.01–50 ng mL^−1^	5.6 pg mL^−1^ (ppt)	[97]
OTA	GATCGGGTGTGGGTGGCGTAAAGGGAGCATCGGACA	Au NPs–rGO	EIS	0.001–50ng mL^−1^	0.3 pg mL^−1^	[98]
OTA	GATCGGGTGTGGGTGGCGTAAAGGGAGCATCGGACA	Au NPs–rGO	EIS	0.1–200 ng mL^−1^	0.03 ng mL^−1^	[99]
OTA	GATCGGGTGTGGGTGGCGTAAAGGGAGCATCGGACA	graphene	DPV	0.001–5 ng mL^−1^	0.13 pgmL^−1^	[100]
